# Re-examining the bad news game: No evidence of improved discrimination of Indian true and fake news headlines

**DOI:** 10.3758/s13423-025-02827-x

**Published:** 2025-12-15

**Authors:** Tina Seabrooke, Ariana Modirrousta-Galian, Philip A. Higham

**Affiliations:** 1 Centre for Perception and Cognition, School of Psychology, University of Southampton, Highfield, Southampton, SO17 1BJ UK; 2 University College London, London, UK

**Keywords:** Psychological inoculation, Misinformation, Fake news, News discernment, Media literacy, Receiver operating characteristic analysis

## Abstract

Gamified inoculation interventions such as the Bad News game are a widely adopted approach to mitigating the influence of misinformation. While Bad News has been predominately studied with participants from Western, Educated, Industrialized, and Rich Democracies (WEIRD), one recent study (Iyengar et al., *Applied Cognitive Psychology,* 37:290–303, 2023) assessed its efficacy in an Indian sample. In that study, participants rated the reliability of a series of Indian news headlines in a pre-test, played Bad News, and completed a post-test with a different set of headlines. Participants showed better discrimination of true and fake headlines in the post-test than the pre-test. This finding contrasts with a meta-analysis showing that Bad News primarily produces a conservative response bias rather than improving discrimination (Modirrousta-Galian and Higham, *Journal of Experimental Psychology: General*, 152:2411–2437, 2023). The current preregistered study used the same design as Iyengar et al., although participants of Indian nationality (*N* = 150) were recruited via Prolific and the allocation of news headlines to the pre-test and post-test was counterbalanced. When both counterbalancing conditions were included, no significant differences in discrimination or response bias appeared between the pre-test and post-test. When only the counterbalancing condition matching Iyengar et al.’s experiment was examined, no significant effect on discrimination was observed, but a conservative response bias shift was seen in the post-test. This finding suggests that the Bad News game may be less effective for improving discrimination than previously thought – an important consideration given its popularity as an intervention to combat misinformation.

**Supplementary Information:**

The online version contains supplementary material available at 10.3758/s13423-025-02827-x.

## Introduction

Misinformation is a global challenge that can undermine democracy, polarize society, and harm public wellbeing. For example, during the early stages of the COVID-19 pandemic, Iran saw a surge in poisonings linked to the false belief that consuming alcohol disinfectant could prevent or treat the virus (Hassanian-Moghaddam et al., 2020). Since misinformation can spread rapidly on social media (Pennycook & Rand, 2021), research has focused on understanding why people share inaccurate content on these digital platforms, and developing targeted interventions that help people to distinguish between real and false information online.

Online gamified interventions have gained traction as a tool for combating misinformation. The Bad News game is a particularly influential example (Roozenbeek & van der Linden, 2019). This free, browser-based game has been played by over one million people (Maertens et al., 2021), received substantial media attention (e.g., BBC News, 2018; Gold, 2019), and been translated into over 20 languages for global use (Lewandowsky & van der Linden, 2021).

Based on Inoculation Theory (McGuire, 1961), Bad News draws on a biological metaphor in which misinformation is considered a virus that spreads by infecting people. By exposing people to a weakened dose of the “virus” (i.e., misinformation), the researchers aim to pre-emptively inoculate players by triggering the creation of “mental antibodies” to combat misinformation. While the content of psychological inoculations is often topic-specific, the Bad News game aims to provide a “broad-spectrum vaccine against misinformation” (Roozenbeek & van der Linden, 2019, p. 2) by highlighting common strategies used to spread misinformation.

When playing Bad News, participants are introduced to common misinformation-spreading techniques by spreading misinformation themselves. Their goal is to gain both credibility and social media followers. Participants learn to adopt six misinformation-spreading techniques, summarized by the acronym DEPICT: “Discrediting opponents, Emotional language use, increasing intergroup Polarization, Impersonating people through fake accounts, spreading Conspiracy theories, and evoking outrage through Trolling” (Roozenbeek et al., 2022, p. 2). During the game, participants are presented with Twitter-like social media posts and make decisions that affect their trajectory in the game. Participants are rewarded when they adopt behaviors consistent with the DEPICT techniques and are encouraged to adopt those behaviors when they do not.

In the first evaluation of Bad News, Roozenbeek and van der Linden (2019) had participants rate the reliability of tweet-like news headlines in a pre-test, play Bad News, and then complete a post-test that was identical to the pre-test. Participants rated fake news headlines as less reliable in the post-test than the pre-test, which was taken as preliminary evidence that the game inoculates players against misinformation. Similar findings have since been reported, boosting the popularity of Bad News (e.g., Basol et al., 2020; Iyengar et al., 2023; Maertens et al., 2021; Roozenbeek et al., 2021, 2022; Traberg et al., 2024).

Importantly, however, these studies examined participants’ mean reliability ratings for true and false news headlines. This approach has received recent criticism because it conflates discrimination (ability to distinguish between true and false news) and response bias (overall tendency to rate all news as true or false). Analyzing mean ratings is particularly problematic when researchers focus on ratings for false news headlines and ignore ratings for true news entirely. However, even when researchers compute *mean difference scores* between reliability ratings for true and false news headlines, discrimination and response bias remain confounded (Higham et al., 2024). As argued previously (Batailler et al., 2022; Guay et al., 2023; Higham et al., 2024; Modirrousta-Galian & Higham, 2023; Modirrousta-Galian et al., 2023; Nahon et al., 2024), we propose that misinformation interventions should target *discrimination* of true and false information. Interventions that increase skepticism of all news – thereby engendering a more conservative response bias – could have harmful consequences. For example, such interventions could reduce belief in both false statements such as “*5G towers cause COVID-19*” and true statements such as “*COVID-19 vaccinations protect against serious illness.*” Such an outcome would be overly general. Furthermore, given that most people encounter more true than false news in their everyday lives (e.g., Grinberg et al., 2019; Guess et al., 2019), an intervention that leads to increased skepticism of true news is likely to have a negative overall effect if scaled.

In a recent meta-analysis, Modirrousta-Galian and Higham (2023) re-analyzed all available data on Bad News using receiver operating characteristic (ROC) analysis, a technique based on signal detection theory (Macmillan & Creelman, 2005) that is designed to separate discrimination and response bias effects. In all but two cases, they found that Bad News produced a conservative response bias but did not improve discrimination of true and false news (for similar recent findings, see Graham et al., 2023; Maertens et al., 2024, Study 3). Table 1 provides a brief overview of these experiments, as well as several experiments that have been published since Modirrousta-Galian and Higham’s paper and have been subjected to ROC analysis. See Appendix A (Table S1) in the Online Supplementary Material (OSM) for a more comprehensive overview of these experiments.

**Table 1:** Brief summary of past effects of Bad News on discrimination and response bias as measured by receiver operating characteristic (ROC) analysis

Experiment	Sample	Control condition?	Different news items?	Counterbalanced?	Main findings	
					Discrimination	Response bias
Roozenbeek and van der Linden (2019)	Online sample, *N* = 14,163–14,266	No	No	N/A	***p* <.001, *d* = 0.17**	***p* <.001, *d* = 0.40**
Basol et al. (2020)	Prolific, *N* = 198	Yes	No	N/A	T: *p* =.359, d = 0.09 C: *p* =.543, d = 0.05	**T: *p* <.001, *d* = 0.47** C: *p* =.090, *d* = 0.12
Maertens et al. (2021), Exp. 1	Prolific, *N* = 118	Yes	No	N/A	T: *p* =.943, *d* = 0.12 C: *p* =.534, *d* = −0.20	**T: *p* <.001, *d* = 1.40** **C: *p* =.002, *d* = 0.55**
Maertens et al. (2021), Exp. 2	Prolific, *N* = 110	Yes	No	N/A	T: *p* =.482, *d* = 0.20 C: *p* =.909, *d* = 0.08	**T: *p* <.001, *d* = 0.87** C: *p* =.541, d = 0.20
Roozenbeek et al. (2021), Exp. 1	Online sample, *N* = 480	No	Yes	Yes	Set A–A: *p* =.074, *d* = 0.16 Set B–B: *p* =.452, *d* = 0.07	**Set A–A: *p* <.001, *d* = 0.39** Set B–B: *p* =.426, *d* = 0.07
Roozenbeek et al. (2022), Exp. 1	Online sample, *N* = 1,216	No	No	N/A	*p* =.448, *d* = 0.02	***p* <.001, *d* = 0.37**
Roozenbeek et al. (2022), Exp. 2	Online sample, *N* = 968	No	Yes	Yes	**Set A–A: *p* =.041, *d* = 0.13** Set B–B: *p* =.437, *d* = 0.05	**Set A–A: *p* =.003, *d* = 0.19** Set B–B: *p* =.185, *d* = −0.08
Graham et al. (2023)	Canadian undergraduates, *N* = 353	Yes	No	N/A	*p* =.70	***p* <.001**
Iyengar et al. (2023)	Adults recruited from Indian universities, *N* = 1,002	No	Yes	No	***p* <.001, *d* = 0.45**	***p* <.001, *d* = 0.15**
Modirrousta-Galian et al. (2023)	Prolific (USA only), *N* = 282	Yes*	N/A	N/A	*F* < 1	X
Axelsson et al. (2024)	Swedish school students, aged 16–19 years, *N* = 516	No	No	N/A	***p* <.001, *d* = 0.06**	X
Leder et al. (2024), Exp. 4	Online sample, *N* = 2,558	Yes**	No	N/A	**T: *p* =.037, *d* = 0.06** C: *p* =.121, *d* = −0.05	**T: *p* <.001, *d* = 0.16** **C: *p* <.001, *d* = 0.35**
Leder et al. (2024), Exp. 5	Online sample, *N* = 419	No	No	N/A	*p* =.134, *d* = 0.06	*p* =.975, *d* = 0.00
Leder et al. (2024), Exp. 6	Online sample, *N* = 882	No	No	N/A	***p* <.001, *d* = 0.19**	***p* =.004, *d* = −0.10**

*Exp*. Experiment, *T* treatment, *C* control, *N/A* not applicable, *X* not available.

Bold emphasis indicates a statistically significant difference between pre-test and post-test.

*In Modirrousta-Galian et al. (2023), participants first completed Bad News, a gamified or non-gamified inductive learning task, or no initial task (control condition), before completing a final test.

** In Leder et al. (2024), Experiment 4, treatment participants played the Bad News game and completed feedback exercises, whereas control participants only played the Bad News game.

In the first case (Roozenbeek & van der Linden, 2019), although discrimination improved in the post-test, the true and false headlines were not comparable. The false headlines were necessarily uncertain because they were created by the researchers. In contrast, the true news headlines had received extensive media coverage and hence would have been unquestionably true for many participants. Indeed, pre-test reliability ratings for these statements approached ceiling, making it impossible to accurately evaluate the true effects of Bad News on discrimination.

In the second case (Iyengar et al., 2023), Bad News improved discrimination even though there was no obvious ceiling effect on the true news. Indian participants completed a pre-test, Bad News, and a post-test. In each test, participants rated the reliability of two true and six fake tweet-like Indian news headlines (with accompanying pictures and different stimuli in each test). The fake items consisted of two headlines each corresponding to the Impersonation, Conspiracy, and Discrediting techniques. The study was conducted remotely, via several enumerators, allowing for a very large sample (*N* = 1,002). Iyengar et al. used mean ratings to measure discrimination performance, while Modirrousta-Galian and Higham (2023) re-analyzed the same data using ROC analysis. With both approaches, participants showed significantly higher discrimination in the post-test than in the pre-test. In the ROC analysis, discrimination improved with a moderate effect size (*d* = 0.45).

The decisive effect of Bad News on discernment makes Iyengar et al.’s (2023) study stand out from the others that have been subjected to ROC analysis. There are three potential reasons for this unusual result. First, Iyengar et al. used Indian news headlines, while the other studies that Modirrousta-Galian and Higham (2023) re-analyzed all used Western news headlines. Therefore, the specific news headlines may be responsible for the different results. Second, Iyengar et al. recruited Indian participants, whereas most gamified inoculation studies recruit participants from predominately Western, Educated, Industrialized, and Rich Democracies (WEIRD). It is therefore possible that population differences are responsible for the different results. Finally, the striking effect observed by Iyengar et al. may reflect an experimental confound: the assignment of news headlines to the pre-test and post-test was not counterbalanced. Enhanced post-test discrimination could, therefore, reflect the post-test headlines being easier to discriminate than the pre-test headlines.

## The current study

We built on Iyengar et al.’s (2023) study while evaluating the possibilities noted above. We first conducted a pilot study, which used the same experimental design and materials as Iyengar et al. but was conducted in-person at the University of Southampton, with 42 students. Given the relatively small and Western sample, this study is reported in Appendix B of the OSM. In our main study, 150 participants of Indian nationality completed the same experiment remotely. Both studies used Iyengar et al.’s pre/post design, but with the pre-test and post-test news headlines counterbalanced across participants. Since Iyengar et al. adopted a large-scale protocol involving 1,002 participants recruited via multiple enumerators from affiliated colleges within their university, it was not possible for an independent research team to fully adopt their methodology. Our work was not intended as a direct replication of Iyengar et al., but rather an extension of their work, using a procedure that was closely matched where possible.

## Method

### Transparency and openness

This study was preregistered. The preregistration, data, analytic code, and materials needed to replicate this study are available on the Open Science Framework (https://osf.io/7xw6a/?view_only=c47138168f0945c29e97ea2f4d5ca2ce). The study was approved by the School of Psychology Ethics Committee at the University of Southampton (ID: 79,617.A2). We report all measures, manipulations, and exclusions in the study.

### Participants

When re-analyzing Iyengar et al.’s (2023) data, Bad News yielded an effect size of *d* = 0.45 on discrimination performance (Modirrousta-Galian & Higham, 2023). Although this is a medium-sized effect, we preregistered that we would recruit enough participants to detect a more conservative small-to-medium effect of *d* = 0.30. This effect size was chosen because it approximately corresponds to the “small” effect sizes observed in Iyengar et al. with mean ratings. A power analysis in G*Power (Faul et al., 2007) suggested that 147 participants were needed to detect an effect of this size with a two-tailed paired-samples *t*-test (*n* = 147, *d*_z_ = 0.30, 1—*β* =.95, *α* =.05). We preregistered that we would recruit 150 participants. Although the sample size was large enough to detect the “small” effects that were observed in Iyengar et al. (*d* = 0.348 and 0.337 for the Impersonation and Conspiracy categories, respectively), it was not large enough to detect the “very small” effect (*d* = 0.125 for Discrediting). Detecting an effect of this size would have required a very large sample and, given its negligible size, detecting it would be of questionable value.

The study was conducted using Qualtrics, which provides a measure of fraud detection. Participants scoring less than.5 on this measure (out of 1) are considered “likely to be bots.” Although performance on this measure was not a preregistered exclusion criterion, we replaced four participants who scored less than.5. We preregistered that that we would exclude participants who completed the study exceptionally fast (more than three standard deviations below the mean), participants who reported technical issues at the end of the study, and any participants who stated that they did not complete the Bad News game. No participants were excluded based on these preregistered criteria.

The final sample consisted of 150 participants (90 male, 59 female, one “prefer not to say”), who were aged between 18 and 40 years (*M* = 29.39, *SD* = 5.85). In total, 56.67% of participants stated that English was their first language and 54.67% of participants stated that their highest education attainment was “Postgraduate/Higher.” The mean political orientation rating across participants was 3.79 (*SD* = 1.26), with ratings of 1 and 7 representing “Very Left-wing” and “Very Right-wing,” respectively. Prolific prescreening restrictions were applied, such that the study was only advertised to participants who were of Indian nationality, spoke fluent English, were aged between 18 and 40 years, and had a minimum Prolific approval rating of 90%.

### Materials

The materials were taken from Iyengar et al. (2023). There were two sets of real Indian news headlines, with each set containing two true news headlines (control items) and six fake headlines. The fake headlines within each set contained two headlines each belonging to the categories *Impersonation*, *Conspiracy*, and *Discrediting*. The allocation of item sets to the pre-test and post-test was counterbalanced across participants, who were randomly assigned to Set A (*N* = 74) or Set B (*N* = 76).

### Procedure

Participants took part in the study remotely via Prolific. Participants provided informed consent, typed their age into a text box, selected their gender (choosing between “Male,” “Female,” “Other,” and “Prefer not to say”), and confirmed whether English was their first-speaking language (choosing between “Yes” and “No”). They also selected their highest educational attainment (choosing between “Undergraduate/Lesser” and “Postgraduate/Higher”) and rated their political ideology on a scale from 1 (*Very Left-wing*) to 7 (*Very Right-wing*).

Participants then completed a pre-test, Bad News, and the post-test.^1^ During the pre-test and post-test, participants were shown six fake and two true news headlines in a random order. They had unlimited time to rate the reliability of each headline using a scale from 1 (*Less reliable*) to 7 (*Most reliable*). After the pre-test, participants were instructed to click a link that directed them to the Bad News game (https://www.getbadnews.com/books/test/) on a new page. Participants were instructed not to complete any inbuilt surveys within the Bad News game. To encourage completion of the Bad News game, participants were only able to progress with the study after at least 7.5 min.

Upon completion, participants completed the post-test and were asked to select the badge that represented “trolling” from the Bad News game.^2^ Participants were also provided with a text box in which they could note any issues that they experienced when completing the study, or if they did not earn all six badges in the Bad News game. Finally, participants received a written debrief, were presented again with the fake news headlines and informed that they were all fake, and were then redirected back to Prolific. On average, participants took approximately 26 min to complete study. However, as progression throughout the study was self-paced, the completion time varied between participants.

### Data analysis approach

We preregistered that we would conduct ROC analysis on participants’ pre-test and post-test reliability ratings. We first calculated pre-test and post-test hit rates (HRs; the proportion of true news items that were correctly identified as true) and false alarms rates (FARs; the proportion of false news items that were incorrectly identified as true) for each participant using each level of the rating scale. We then created pre-test and post-test ROC curves by plotting the average HR as a function of the average FAR for each scale point.

The pre-test and post-test ROC curves can be compared to a hypothetical straight diagonal line that runs from the [0,0] to [1,1] coordinates, which corresponds to chance-level discrimination. Bowing of the ROC curves, towards the top-left corner of the plot, indicates above-chance discrimination. The further the bowing, the better the discrimination. We quantified discrimination by calculating the area under the curve (AUC) for each participant, using the trapezoidal rule formula (Pollack & Hsieh, 1969). AUC values range between 0 and 1, with.5 and 1 reflecting chance-level and perfect discrimination, respectively.

Response bias can similarly be visualized by evaluating the position of the points (HR/FAR pairs) on the ROC curves. If the points cluster toward the top-right of the curve, the HRs and FARs will both be high, suggesting liberal responding (i.e., willingness to use high scale values). If they cluster toward the bottom-left of the curve, both the HRs and FARs will tend to be low, suggesting conservative responding (i.e., unwillingness to use high scale values). To quantify response bias, we calculated *B"*_D_ for each scale point and averaged the values to give an overall estimate of liberal or conservative responding (Donaldson, 1992). *B"*_D_ varies between −1 and + 1, with −1 reflecting extremely liberal responding (strong tendency to rate all items as true) and + 1 reflecting extremely conservative responding (strong tendency to rate all items as false). If *B"*_D_ = 0, there is no response bias. For a more complete explanation of ROC analysis for misinformation research, see Higham et al. (2024).

We compared participants’ pre-test and post-test AUC and *B"*_D_ scores using repeated-measures *t*-tests and Bayes factors (*BF*_10_). *BF*_10_ values were calculated using version 0.9.12.4.7 of the *BayesFactor* package (Morey & Rouder, 2024) in RStudio (version 4.3.2) and interpreted according to the evidence categories outlined by Lee and Wagenmakers (2013). We conducted a preregistered analysis of the complete dataset and non-preregistered analyses of each counterbalancing condition. Finally, to facilitate comparison with Iyengar et al. (2023), a non-preregistered analysis of the mean ratings are provided in Appendix C of the OSM. We did not follow the precise approach taken by Iyengar et al. because further inspection of their analytic code, as well as the degrees of freedom reported in the paper, suggests they conducted several between-subjects tests on their pre/post data. We were able to replicate their *t*-test statistics with between-subjects tests, but not with repeated-measures tests (Table S2, OSM). Importantly, the repeated-measures analyses did not change the overall pattern of results: relative to pre-test reliability ratings, post-test reliability ratings increased for the true news headlines (Control) and decreased for the false news headlines (Conspiracy, Discrediting, and Impersonation). To facilitate comparison with this result, we also analyzed the mean ratings from our study with four repeated-measures *t*-tests (Table S3, OSM). No significant differences were observed.

## Results

**Fig. 1:**
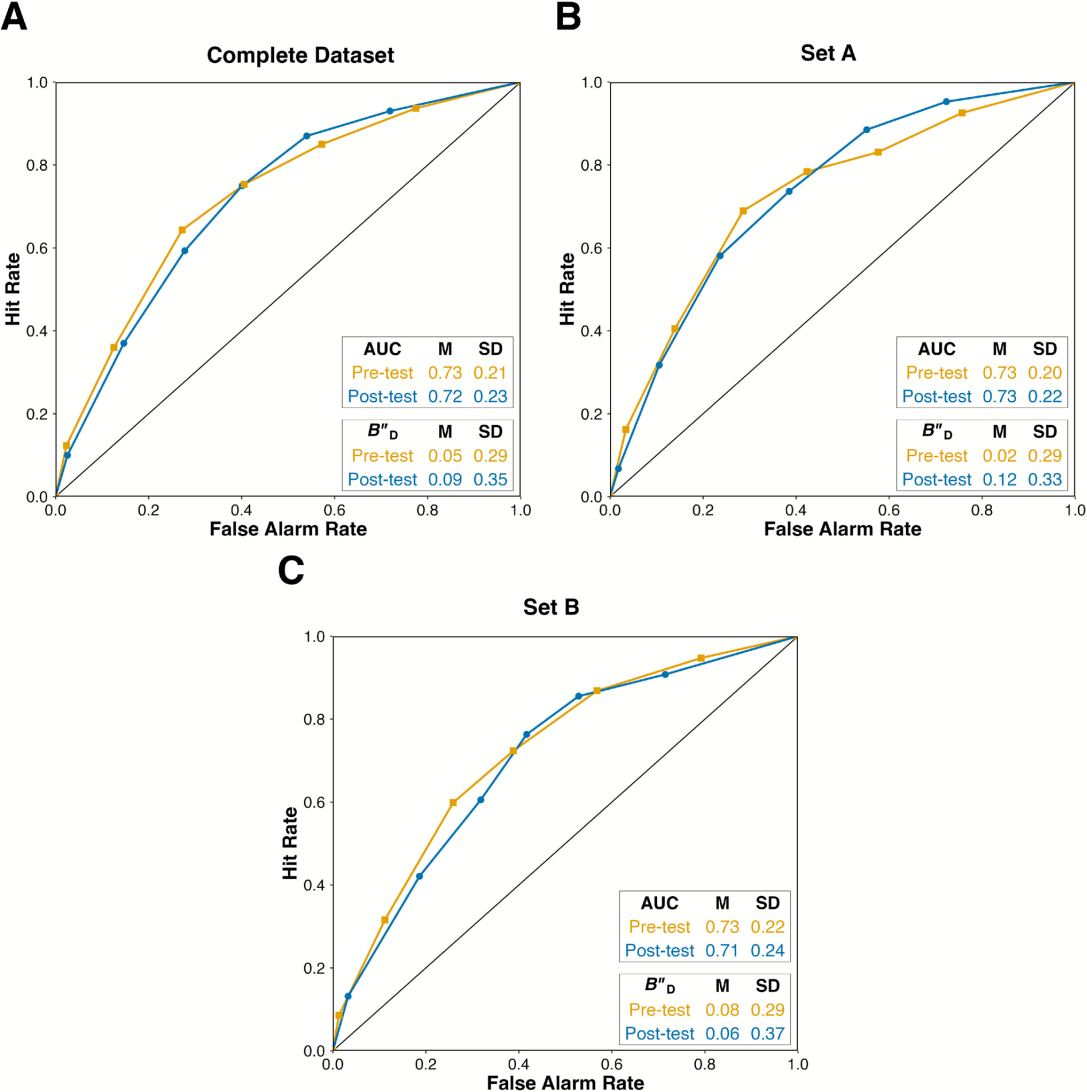
Receiver operating characteristic (ROC) curves for the pre-test and post-test reliability ratings

Figure 1A displays the pre-test and post-test ROC curves, and descriptive statistics, for all participants. Figures 1B and C show the equivalent information for participants allocated to Set A and Set B, which correspond to the two counterbalancing conditions used for assigning news headlines in the pre-test and post-test. Set A corresponds to the pre-test and post-test stimuli that were used in Iyengar et al. (2023).

### Complete dataset (preregistered)

When considering all participants (Fig. 1A; *N* = 150), paired-sample *t*-tests showed that neither discrimination (AUC), *t*(149) = 0.49, *p* =.63, *d*_z_ = 0.04, *BF*_10_ = 0.10, nor response bias (*B"*_D_), *t*(149) = 1.23, *p* =.22, *d*_z_ = 0.10, *BF*_10_ = 0.19, significantly differed between the pre-test and post-test. These results contrast with Modirrousta-Galian and Higham’s (2023) re-analysis of Iyengar et al. (2023), where participants showed better discrimination and a more conservative response bias in the post-test than the pre-test.^3^

### Subset analyses (non-preregistered)

We counterbalanced the allocation of news headlines to the pre-test and post-test, a step not included in Iyengar et al. (2023), to address potential item-related confounds. To test whether this difference underpinned the discrepant results, we conducted two 2 (test: pre-test vs. post-test) × 2 (counterbalancing condition: Set A vs. Set B) mixed analyses of variance (ANOVAs), one on discrimination and the other on response bias scores. Set A participants (Fig. 1B; *N* = 74) saw the images in the same order as Iyengar et al.’s participants, while Set B participants (Fig. 1C; *N* = 76) saw the images in the reverse order. With respect to discrimination, AUC scores did not significantly differ between the pre-test (*M* =.73, *SD* =.21) and post-test (*M* =.72, *SD* =.23), as revealed by the non-significant main effect of test, *F*(1, 148) = 0.23, *p* =.63, η_g_^2^ <.001, *BF*_10_  ≈ 0.14. Likewise, the non-significant main effect of counterbalancing condition confirmed that AUC scores did not significantly differ for Set A (*M* =.73, *SD* =.21) and Set B (*M* =.72, *SD* =.23) participants, *F*(1, 148) = 0.17, *p* =.68, η_g_^2^ <.001, *BF*_10_ ≈ 0.19. Finally, no significant interaction was observed, *F*(1, 148) = 0.57, *p* =.45, η_g_^2^ =.001, *BF*_10_ ≈ 0.27. This analysis – particularly the lack of improved discrimination in the post-test for Set A participants – does not replicate the pattern observed in Modirrousta-Galian and Higham’s (2023) re-analysis of Iyengar et al.

With respect to response bias (*B"*_D_), no significant main effects of test, *F*(1, 148) = 1.59, *p* =.21, η_g_^2^ =.003, *BF*_10_ ≈ 0.26, or counterbalancing condition, *F*(1, 148) = 0.01, *p* =.91, η_g_^2^ <.001, *BF*_10_ ≈ 0.17, were observed. *B"*_D_ scores did not significantly differ between the pre-test (*M* =.05, *SD* =.29) and post-test (*M* =.09, *SD* =.35), or between Set A (*M* =.07, *SD* =.32) and Set B (*M* =.07, *SD* =.33) participants. The interaction approached significance, *F*(1, 148) = 3.67, *p* =.06, η_g_^2^ =.008, although the Bayes factor was inconclusive, *BF*_10_ ≈ 0.97.

For completeness, we separately assessed the effects of test in each counterbalancing condition. Set A participants (Fig. 1B) showed a significantly more conservative response bias in the post-test than the pre-test, *t*(73) = 2.18, *p* =.03, *d*_z_ = 0.25, although the Bayes factor provided only anecdotal evidence for the alternative hypothesis, *BF*_10_ = 1.17. Thus, Set A participants in our study and Iyengar et al.’s (2023) participants were somewhat more skeptical of the post-test headlines than the pre-test headlines, perhaps not because those headlines appeared after participants played Bad News, but rather because those headlines seemed inherently less reliable (i.e., item effects). If this is the case, then Set B participants – who saw the original post-test headlines in the pre-test and the original pre-test headlines in the post-test – should show a post-test increase in *liberal* responding. Contrary to this prediction, Set B participants (Fig. 1C) showed no significant difference between their pre-test and post-test *B"*_D_ scores, *t*(75) = 0.48, *p* =.63, *d*_z_ = 0.05, *BF*_10_ = 0.14. Hence, the increase in post-test conservative responding among Set A participants may reflect a more complex influence of the specific headlines that participants rated in the post-test.^4^

## Discussion

In this preregistered study, we examined Indian participants’ ability to discriminate between true and fake Indian news headlines before and after playing Bad News. We followed Iyengar et al.’s (2023) study design, while counterbalancing the news headlines in the pre-test and post-test. When collapsing across counterbalancing conditions, no significant differences were seen between the pre-test and post-test in discrimination or response bias. When only the counterbalancing condition corresponding to Iyengar et al.’s procedure was considered, we observed a conservative response bias shift in the post-test (with indecisive evidence from the Bayes Factor), but no significant effect on discrimination.

Our results partially align with previous findings. Iyengar et al. (2023) observed an increase in discrimination after playing Bad News, even when ROC analysis was used (Modirrousta-Galian & Higham, 2023). This finding diverged from the existing literature, where participants do not typically show improved discrimination after playing Bad News (see Table 1). Our discrimination results are consistent with the broader literature, but not Iyengar et al.’s findings.

Modirrousta-Galian and Higham’s (2023) re-analysis further concluded that, consistent with their overall meta-analysis (see also Table 1), Iyengar et al.’s (2023) participants showed an increase in conservative responding after playing Bad News. In our study, we only replicated this result when analyzing just the counterbalancing condition consistent with Iyengar et al.’s study. Thus, both this result and Iyengar et al.’s response bias shift may be due to item effects, underscoring the importance of counterbalancing in pre/post designs.

Although the lack of improved discrimination after playing Bad News in our study is consistent with the overall consensus from Modirrousta-Galian and Higham’s (2023) meta-analysis (see also Graham et al., 2023; Maertens et al., 2024, Study 3), it sits less well with Lu et al.’s (2023) meta-analysis. Lu et al. reported that psychological inoculation improves discernment between true information and misinformation. However, their meta-analysis included both gamified and non-gamified interventions. Modirrousta-Galian and Higham, by contrast, focused only on the gamified interventions Bad News and GoViral! and concluded that they produce conservative response bias shifts rather than improved discernment. Thus, it is possible that the wider range of studies analyzed by Lu et al. is responsible for this discrepancy. Interestingly, when considering just the studies that were re-analyzed in both meta-analyses, Lu et al. also appeared to find largely negative effects of psychological inoculation on real news credibility ratings, which would be consistent with a conservative response bias shift. Hence, when comparable datasets are considered, the results of Modirrousta-Galian et al. and Lu et al. are more similar than they first appear.

One further study by Axelsson et al. (2024) warrants discussion. In a Swedish classroom study, the authors found that discrimination of true and fake news items was better after playing Bad News than before (mean pre-test AUC =.87 vs. mean post-test AUC =.91). While this difference was statistically significant (*p* <.001), the effect (*d* = 0.06) was far below the conventional benchmark for a small effect (*d* = 0.20). Our intervention effect on discrimination was comparable (*d*_z_ = 0.04), but we reached the opposite conclusion to Axelsson et al. because our pre-test and post-test discrimination scores did not significantly differ. With very large samples, studies are more likely to detect very small effects (Schäfer & Schwarz, 2019). As Combs (2010) noted, even correlations that round off to zero (*r* =.0043) are statistically significant given a sufficiently large (212,014) number of observations. Such small effects may be of questionable practical or theoretical value.

Iyengar et al. (2023) also used a large sample of 1,002 participants, aligning with current trends in misinformation research (Kiili et al., 2024). In contrast, we recruited 150 participants based on a smaller discrimination effect than Iyengar et al. found. It is possible that we too would have a seen a post-test improvement with a larger sample. Given the substantial resources needed for large-scale replications, researchers should carefully consider whether the effects are meaningful enough to justify them.

### Improving discernment of true and fake news

Why doesn’t Bad News improve discrimination? We discuss three possibilities. First, Bad News focuses primarily on spotting misinformation, with little emphasis on spotting true news, but discrimination requires both (Maertens et al., 2024). Second, the features highlighted in the game may not uniquely predict fake news. For example, Hart et al. (2020) found that reputable newspapers provided highly polarized news coverage – one of the misinformation techniques highlighted in Bad News – questioning whether polarization uniquely predicts misinformation.

Finally, Bad News focuses on explicit rules and features to identify fake news. However, without any training, participants show reasonable discrimination by using tacit knowledge. Modirrousta-Galian et al. (2024) had participants identify the strategy they used when judging the veracity of true and fake news headlines. Participants showed above-chance discrimination performance, even though they indicated they were guessing or using intuition for most news headlines. Training procedures that encourage tacit knowledge, such as inductive learning training, may be more effective (Modirrousta-Galian et al., 2024). Interestingly, Seabrooke et al. (2025) found inductive learning markedly improved people’s discrimination of real and AI-generated images. Leder et al. (2024) also recently reported that adding feedback exercises after the Bad News game was useful for improving discrimination. Feedback is a key component of inductive learning regimes and a highly effective tool for improving category and concept learning (Ashby et al., 2002).

### Limitations and future directions

To facilitate comparison with Iyengar et al. (2023), we adopted their broad methodology, which is suboptimal in several ways. First, we used Iyengar et al.’s set of headlines. Not only did this mean that the news headlines were older than in the original study, but we were also limited to their stimuli. Iyengar et al. only presented two true and six fake news headlines in the pre-test and post-test. While these numbers are comparable with many other Bad News experiments (e.g., Roozenbeek & van der Linden, 2019; see Table S1, OSM), the small numbers of items used is a major limitation of the paradigm, and using more news headlines would provide a better representation of the game’s effect (Graham et al., 2023; Modirrousta-Galian et al., 2023). Indeed, it is possible that the variability in results that has been observed across studies is attributable to the small number of items used. As a case in point, we argued in the *Introduction* that the improved discrimination performance seen after participants played Bad News in Roozenbeek and van der Linden (2019) is likely due to a ceiling effect on the two true news items used in that study. In general, we think it is preferable to test the effects of an intervention against a wide range of stimuli, rather than carrying a very small number stimuli across experiments, even if those stimuli have previously produced reliable effects or have been psychometrically validated.

We also adopted Iyengar et al.’s (2023) pre/post design, which lacked a control condition – another major limitation of the paradigm. While pre/post designs are common in the literature (see Table 1), they are suboptimal because participants may respond differently in the pre-test and post-test for reasons other than the intervention (e.g., fatigue or experience with news headlines in general). Several previous studies have adopted both a pre/post design *and* a control condition in which participants typically play Tetris for the time it takes treatment participants to play the Bad News game (see Table S1, OSM). These experimental designs are certainly better than the pure pre/post design used in Iyengar et al. and here, but they are also not optimal. Tetris and Bad News differ in multiple ways, not least in terms of exposure to misinformation, which makes it difficult to determine the specific effect of the intervention. Indeed, at least one experiment found that participants’ discrimination performance improved after playing Tetris, although this pattern was likely attributable to item effects (Basol et al., 2021; data re-analyzed by Modirrousta-Galian & Higham, 2023). Ideally, Bad News should be compared to a control condition in which participants see the same material and the gamification elements but critically do not receive training on spotting fake news.

Finally, we propose two avenues for future research. First, we only recruited participants who were of Indian nationality; it would be fruitful to directly compare participants from Western and non-Western nationalities to examine cultural differences in misinformation perception and detection. Second, with a greater range of headlines, it would be possible to systematically vary the topic and framing of headlines to investigate whether (and why) certain headlines are easier to discriminate as true or false than others.

## Conclusion

Overall, our study is consistent with a growing literature suggesting that, when ROC analysis is used to separate discrimination and response bias, Bad News often produces a more conservative response bias but does not usually improve discrimination of true and fake news. This consensus is at odds with two recent studies: Axelsson et al. (2024) observed an effect on discrimination, while Leder et al. (2024) reported that adding feedback exercises to the Bad News game enhanced discrimination. However, in both cases, the effect sizes were negligible. Further research is needed to examine whether such discrimination can be enhanced, and whether approaches like inductive learning offer a fruitful alternative.

## Supplementary Information

Below is the link to the electronic supplementary material.

Supplementary file1 (DOCX 485 kb)

## Data Availability

The data and materials needed to replicate this study are available on the Open Science Framework (https://osf.io/7xw6a/?view_only=c47138168f0945c29e97ea2f4d5ca2ce).

## Footnotes

1 As a “living intervention,” Bad News is updated periodically. Our participants completed the version available between 3 May 2024 and 18 May 2024.

2 We included this exploratory question to see how well participants remembered the details of the Bad News game. We did not intend to use this question as a formal attention check because the question was asked at the end of the post-test and was therefore likely to be affected by memory. The question was correctly answered by 75% of participants.

3 We conducted two additional, non-preregistered *t*-tests that repeated these analyses for only the 75% of participants who selected the correct badge for “trolling” at the end of the experiment. Again, neither discrimination performance,* t*(111) = 0.05, *p* =.96, *d*_z_ = 0.005, *BF*_10_ = 0.10, nor response bias scores, *t*(111) = 0.81, *p* =.42, *d*_z_ = 0.08, *BF*_10_ = 0.14, significantly differed between the pre-test and post-test. See Appendix D, Table S4 (OSM) for the descriptive statistics.

4 In a final analysis, we examined participants’ pre-test and post-test responses with the same set of stimuli. We achieved this by comparing Set A participants’ pre-test responses to Set B participants’ post-test responses, and vice versa. No significant effects were observed. These analyses are reported in Appendix E of the OSM.

## Code availability

The code needed to replicate the analyses reported in this study are available on the Open Science Framework (https://osf.io/7xw6a/?view_only=c47138168f0945c29e97ea2f4d5ca2ce).

## Declarations

### Open Practices Statement

This study was preregistered prior to data collection. The timestamped preregistration, data, analytic code, and materials needed to replicate this study are available on the Open Science Framework (https://osf.io/7xw6a/?view_only=c47138168f0945c29e97ea2f4d5ca2ce).
