# Supplementary material for: Re-examining the bad news game: No evidence of improved discrimination of Indian true and fake news headlines
Source: Psychon Bull Rev. 2025 Dec 15;33(1):13. doi: 10.3758/s13423-025-02827-x (PMC12705795; doi:10.3758/s13423-025-02827-x)
Supplement: Supplementary file 1 — Supplementary file1 (DOCX 485 kb) [file 13423_2025_2827_MOESM1_ESM.docx]

# Re-Examining the Bad News Game: No Evidence of Improved Discrimination of Indian True and Fake News Headlines

Tina Seabrooke^1^, Ariana Modirrousta-Galian^1,2^, and Philip A. Higham^1^

^1^ University of Southampton

^2^ University College London

# Supplemental Materials

# Appendix A: Detailed Overview of Previous Experiments

**Table 1**

*Detailed Summary of Past Effects of Bad News on Discrimination and Response Bias as Measured by ROC Analysis*

| Experiment | Population Sample | Key Task Details | Main Findings | |
| --- | --- | --- | --- | --- |
|  |  |  | Discrimination | Response Bias |
| Roozenbeek and van der Linden (2019) | Online convenience sample recruited from the Bad News platform, *N* = 14,163–14,266. | Pre-test/post-test design with all participants playing Bad News between the pre-test and post-test. The same 2 true and 3 false news items were presented in each test. | Improved discrimination from pre-test to post-test, with a very small effect size but extreme Bayes Factor evidence for the alternative hypothesis (*p* < .001, *d* = 0.17, *BF*_10_ = 1.73 × 10^122^). | Conservative response bias shift from pre-test to post-test (*p* < .001, *d* = 0.40, *BF*_10_ = 1.34 × 10^486^). |
| Basol et al. (2020) | Prolific, *N* = 198. | Pre-test/post-test design with 2 conditions: treatment (Bad News) and control (Tetris). Participants rated the same 3 true and 18 false news items in the pre-test and post-test. | Treatment condition: No significant difference in discrimination performance at pre-test and post-test (*p* = .359, *d* = 0.09, *BF*_10_ = 0.17).  Control condition: No significant difference in discrimination performance at pre-test and post-test (*p* = .543, *d* = 0.05, *BF*_10_ = 0.13). | Treatment condition: Conservative response bias shift from pre-test to post-test (*p* < .001, *d* = 0.47, *BF*_10_ = 48,915.96).  Control condition: No significant response bias shift from pre-test to post-test (*p* = .090, *d* = 0.12, *BF*_10_ = 0.45). |
| Maertens et al. (2021), Experiment 1 | Prolific, *N* = 118. | Pre-test/post-test design with 2 conditions: treatment (Bad News) and control (Tetris). At Time Point 1 (T1), participants completed the pre-test and played Bad News / Tetris, followed by a post-test (T2). Participants completed further post-tests 1 week (T3), 5 weeks (T4), and 13 weeks (T5) after T1. In each test, participants rated the same 3 true and 18 false news items. | Treatment condition: No significant effect of time point on discrimination performance (*p* = .943, *d* = 0.12, *BF*_10_ = 0.01).  Control condition: No significant effect of time point on discrimination performance (*p* = .534, *d* = -0.20, *BF*_10_ = 0.03). | Treatment condition: conservative response bias shift from the pre-test to post-tests (*p* < .001, *d* = 1.40, *BF*_10_ = 5.32 × 10^15^).  Control condition: conservative response bias shift from the pre-test to post-tests (*p* = .002, *d* = 0.55, *BF*_10_ = 7.81). |
| Maertens et al. (2021), Experiment 2 | Prolific, *N* = 110. | Pre-test/post-test design with 2 conditions: treatment (Bad News) and control (Tetris). At Time Point 1 (T1), participants completed the pre-test and played Bad News / Tetris, followed by a post-test (T2). Participants completed a further post-test 9 weeks later (T3). In each test, participants rated the same 3 true and 18 false news items. | Treatment condition: No significant effect of time point on discrimination performance (*p* = .482, *d* = 0.20, *BF*_10_ = 0.12).  Control condition: No significant effect of time point on discrimination (*p* = .909, *d* = 0.08, *BF*_10_ = 0.06). | Treatment condition: conservative response bias shift from the pre-tests to post-tests (*p* < .001, *d* = 0.87, *BF*_10_ = 289.85).  Control condition: no significant effect of time point on response bias (*p* = .541, *d* = 0.20, *BF*_10_ = 0.10). |
| Roozenbeek et al. (2021), Experiment 1 | Online convenience sample recruited from the Bad News platform, *N* = 480. | Pre-test/post-test design with all participants playing Bad News between the pre-test and post-test. Half the participants rated news items from Set A in the pre-test and news items from Set B in the post-test. The remaining participants rated Set B items in the pre-test and Set A items in the post-test. Each set contained 2 real and 6 false news items. | Set A–A: No significant difference in discrimination performance in the pre-test and post-test (*p* = .074, *d* = 0.16, *BF*_10_ = 0.48).  Set B–B: No significant difference in discrimination performance in the pre-test and post-test (*p* = .452, *d* = 0.07, *BF*_10_ = 0.13). | Set A–A: conservative response bias shift from pre-test to post-test (*p* < .001, *d* = 0.39, *BF*_10_ = 764.51).  Set B–B: no significant response bias shift from pre-test to post-test (*p* = .426, *d* = 0.07, *BF*_10_ = 0.14). |
| Roozenbeek et al. (2022), Experiment 1 | Online convenience sample recruited from the Bad News platform, *N* = 1,216. | Pre-test/post-test design with all participants playing Bad News between the pre-test and post-test. Participants rated the same 2 true and 7 false news items in each test. | No significant difference in discrimination performance in the pre-test and post-test (*p* = .448, *d* = 0.02, *BF*_10_ = 0.04). | Conservative response bias shift from pre-test to post-test (*p* < .001, *d* = 0.37, *BF*_10_ = 3.53 × 10^26^). |
| Roozenbeek et al. (2022), Experiment 2 | Online convenience sample recruited from the Bad News platform, *N* = 968. | Pre-test/post-test design with all participants playing Bad News between the pre-test and post-test. Half the participants rated news items from Set A in the pre-test and news items from Set B in the post-test. The remaining participants rated Set B items in the pre-test and Set A items in the post-test. Each set contained 4 true and 4 false news items. | Set A–A: A significant increase in discrimination performance from pre-test to post-test (*p* = .041), with a very small effect size (*d* = 0.13) and anecdotal support for the null hypothesis (*BF*_10_ = 0.56).  Set B–B: No significant increase in discrimination performance from pre-test to post-test (*p* = .437, *d* = 0.05, *BF*_10_ = 0.10). | Set A–A: A conservative response bias shift from pre-test to post-test, with a very small effect size (*p* = .003, *d* = 0.19, *BF*_10_ = 5.06).  Set B–B: No significant response bias shift from pre-test to post-test (*p* = .185, *d* = -0.08, *BF*_10_ = 0.17). |
| Graham et al. (2023) | Undergraduates from a Canadian university, *N* = 353. | Four parallel experiments with a pre-test/post-test design and 2 conditions: treatment (Bad News) and control (Tetris). Participants rated the same 8 true and 24 false news items in the pre-test and post-test. | No significant effect of test time (pre-test vs. post-test), condition (Bad News vs. Tetris), or interaction on discrimination performance (smallest *p* = .70), with moderate evidence against a difference between the treatment and control conditions (*BF*_01_ = 8.43). | Significant main effects of test time and condition, qualified by a significant interaction (*p* < .001, *η*_g_^2^ = .03). There was a conservative response bias shift from pre-test to post-test for both conditions, but this pattern was stronger in the Bad News condition. |
| Iyengar et al. (2023) | Adults recruited from Indian universities, *N* = 1002. | Pre-test/post-test design with all participants playing Bad News between the pre-test and post-test. Different news items (2 true, 6 false) were presented in the pre-test and post-test. The sets were not counterbalanced. | Improved discrimination performance from pre-test to post-test (*p* < .001, *d* = 0.45, *BF*_10_ = 5.26 × 10^27^). | A conservative response bias shift from pre-test to post-test, with a very small effect size (*p* < .001, *d* = 0.15, *BF*_10_ = 2,371.10). |
| Modirrousta-Galian et al. (2023) | Prolific (USA only), *N* = 282. | Initial task (Bad News, gamified or non-gamified inductive learning), followed by a final test. A control group completed the final test only. In the final test, participants rated 18 true and 18 false headlines. | No significant effect of condition on discrimination performance (*F* < 1, *BF*_10_ = 0.026). | Not available. |
| Axelsson et al. (2024) | Swedish school students, aged 16-19 years, *N* = 516. | Quasi-experimental pre-test/post-test design, with participants playing Bad News either individually, in pairs, or collaboratively with a class leaderboard, between the pre-test and post-test. Participants rated the same 2 true and 6 false news items in the pre-test and post-test. | A significant improvement in discrimination performance from pre-test to post-test (*p* < .001), with a negligible effect size (*d* = 0.06). | Not available. |
| Leder et al. (2024), Experiment 4 | Online convenience sample recruited from the Bad News platform, *N* = 2,558. | Pre-test/post-test design with participants completing one of two conditions. Participants completed a pre-test, played Bad News, and completed a post-test. After playing Bad News but before the post-test, participants in a feedback + manipulation technique condition were shown 4 news headlines, indicated whether they thought each headline was misleading and explained why, and then received positive or negative feedback. The remaining participants (game only condition) progressed directly from the Bad News game to the post-test. Participants rated the same 2 true and 6 false news items in the pre-test and post-test. | Feedback + manipulation technique condition: significant increase in discrimination performance from the pre-test to post-test (*p* = .037), with a negligible effect size (*d* = 0.063) and moderate support for the null hypothesis (*BF*_10_ = 0.320 ± .07%).  Game only condition: no significant difference in discrimination performance in the pre-test and post-test (*p* = .121, *d* = -0.045, *BF*_10_ = 0.121 ± .19%). | Feedback + manipulation technique condition: conservative response bias shift from pre-test to post-test, with a significant but very small effect size (*p* < .001, *d* = 0.161, *BF*_10_ = 54,979.94).  Game only condition: Conservative response bias shift from pre-test to post-test (*p* < .001, *d* = 0.345, *BF*_10_ = 2.945 × 10^25^). |
| Leder et al. (2024), Experiment 5 | Online convenience sample recruited from the Bad News platform, *N* = 419. | Same design as Leder et al. (2024), Experiment 4, except the game only condition was omitted and different news items (4 real, 4 false) were used in the pre-test and post-test. | No significant difference in discrimination performance in the pre-test and post-test (*p* = .134, *d* = 0.06, *BF*_10_ = 0.167 ± .13%). | No significant difference in response bias in the pre-test and post-test (*p* = .975, *d* = 0.002, *BF*_10_ = 0.055 ± .39%). |
| Leder et al. (2024), Experiment 6 | Online convenience sample recruited from the Bad News platform, *N* = 882. | Same design as Leder et al. (2024), Experiment 5, except participants rated a different set of news headlines (4 real, 4 false) in the pre-test and post-test. | Discrimination performance increased from the pre-test to post-test, with a significant but very small effect size (*p* < .001, *d* = 0.185, *BF*_10_ = 400398.5). | Participants showed a small conservative response bias in the pre-test. This response bias reduced in the post-test, with a significant but negligible effect size (*p* = .004, *d* = -0.097, *BF*_10_ = 2.027 ± .01%). |

*Note*. ROC = Receiver operating characteristic. The main findings were derived from Modirrousta-Galian and Higham (2023), except for Axelsson et al. (2024), Graham et al. (2023), Leder et al. (2024), and Modirrousta-Galian et al. (2023), where the ROC analyses were reported in the original manuscripts. For Roozenbeek and van der Linden (2019), the final sample for Modirrousta-Galian and Higham’s re-analysis was *N* = 13,564. Roozenbeek and van der Linden also recruited participants to rate a fourth false news item in a separate data collection phase, but these data were not reanalyzed by Modirrousta-Galian and Higham. The improved discrimination seen in Roozenbeek and van der Linden is likely due to a ceiling effect on the true news items. Maertens et al. (2021), Experiment 3, and Leder et al., Experiments 1 and 2, were not included because the data from these studies have not been analyzed using ROC analysis. Leder et al., Experiment 3, was not included because participants played the Harmony Square game rather than Bad News. Modirrousta-Galian and Higham did not reanalyze the A–B and B–A comparisons in Roozenbeek et al. (2021), Experiment 1, due to item effects observed in that study. For the same reason, Modirrousta-Galian and Higham also did not reanalyze the conditions that involved the Bad News game in Roozenbeek et al. (2021), Experiment 2. This study is therefore not reported here.

# Appendix B: Pilot Study

## Method

The method was the same as the main study reported in the manuscript, except in the following respects: (1) the study was not preregistered; (2) different participants were recruited (see below); (3) the study was conducted face-to-face in a laboratory cubicle; (4) participants were not required to wait at least 7.5 min before starting the post-test, because the experimenter verified that each participant completed the Bad News game before progressing; (5) participants did not receive a textbox to note any issues, because such issues could be discussed in-person with the experimenter; (6) participants were not re-presented with the fake news headlines at the end of the study; and (7) the study was completed using the online version of the Bad News game (<https://www.getbadnews.com/books/test/>) that was available between 07/03/2023 and 27/03/2023.

### Participants

Forty-two University of [blinded] undergraduate students participated for course credit and the chance to win one of four £25 Amazon vouchers. The sample size was determined by participant availability. A post-hoc sensitivity analysis conducted in G*Power (Faul et al., 2007) confirmed that the study had 81% power to detect an effect of the size observed by Iyengar et al. [(2023)](#_bookmark22) on discrimination (AUC) with a two-tailed paired samples *t*-test (*N* = 42, *d*_z_ = 0.45, *α* = .05).

The participants (37 females, 5 males) were aged between 18 and 30 years (*M* = 19.14, *SD* = 1.92), with 85.71% stating that English was their first language and all participants confirming that their highest education attainment was “Undergraduate/Lesser”. Participants’ mean political orientation rating was 3.17 (*SD* = 1.25), with ratings of 1 and 7 representing “Very Left-wing” and “Very Right-wing”, respectively.

## Results

ROC analysis was conducted on participants’ pre-test and post-test reliability ratings. Figure S1 shows the ROC curves for each test. A paired-samples *t*-test showed that AUC scores did not significantly differ between the pre-test (*M* = .76, *SD* = .19) and post-test (*M* = .80, *SD* = .20), *t*(41) = 1.22, *p* = .23, *d*_z_ = 0.19, *BF*_10_ = 0.33. *B"*_D_ values also did not significantly differ between the pre-test (*M* = .11, *SD* = .28) and post-test (*M* = .18, *SD* = .32), *t*(41) = 1.51, *p* = .14, *d*_z_ = 0.23, *BF*_10_ = 0.48.

## Figure S1

*ROC Curves for the Pre-Test and Post-Test Reliability Ratings in the Pilot Study*

*
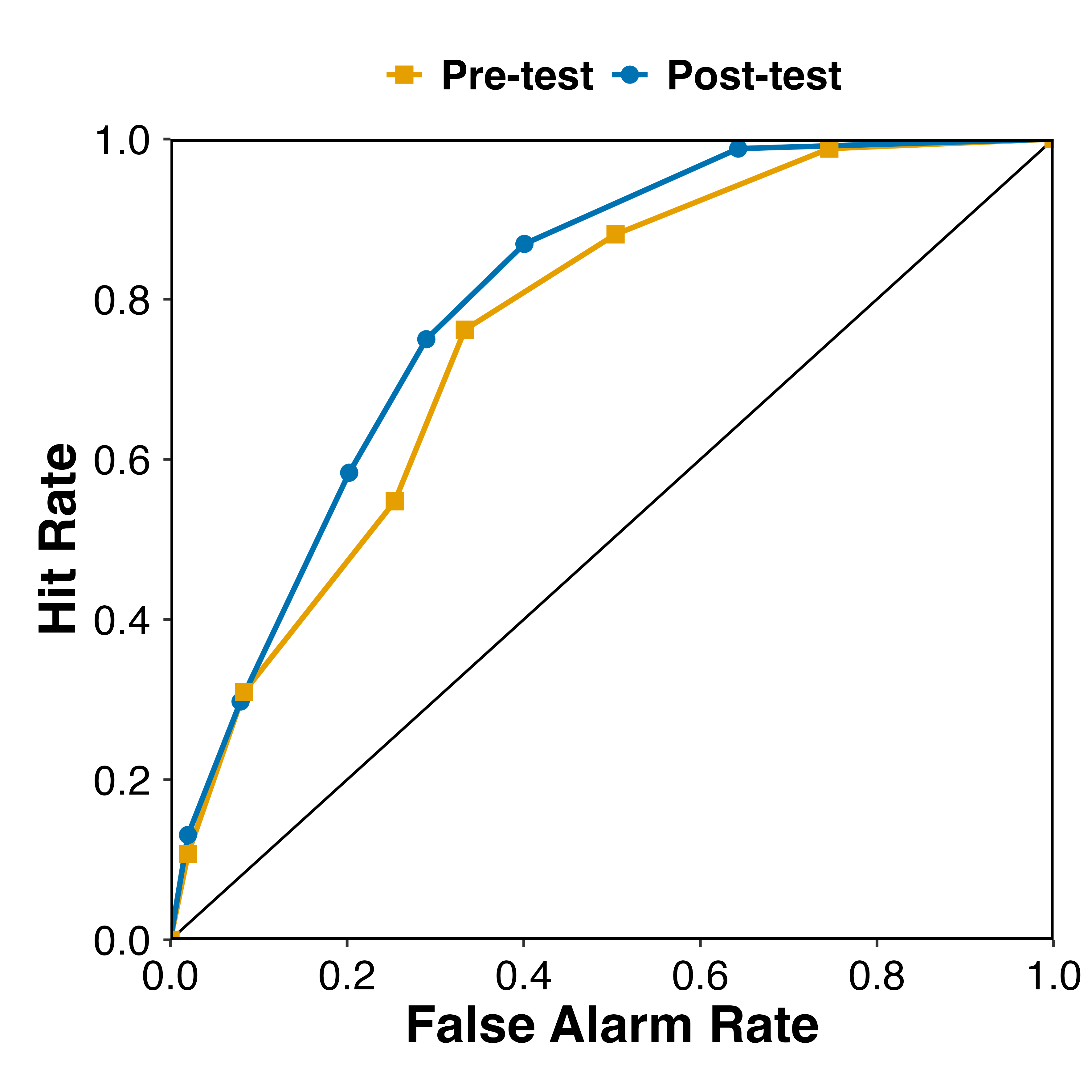
*

*Note*. ROC = receiver operating characteristic.

# Appendix C: Mean Ratings

**Table S2**

*Summary of Mean Reliability Ratings in Iyengar et al. (2023)*

| Headline Type | Pre-test | | Post-test | | *t* | *p* | *d*_z_ | *BF_10_* |
| --- | --- | --- | --- | --- | --- | --- | --- | --- |
|  | *M* | *SD* | *M* | *SD* |  |  |  |  |
| Conspiracy | 3.77 | 1.38 | 3.27 | 1.49 | 10.67 | < .001 | 0.34 | 7.95 × 10^21^ |
| Control | 4.43 | 1.27 | 4.68 | 1.28 | 5.41 | < .001 | 0.17 | 6.22 × 10^4^ |
| Discrediting | 3.54 | 1.44 | 3.36 | 1.39 | 3.97 | < .001 | 0.13 | 85.63 |
| Impersonation | 3.95 | 1.30 | 3.36 | 1.49 | 11.00 | < .001 | 0.35 | 1.20 × 10^23^ |

*Note.* *M* and *SD* represent mean and standard deviation, respectively. Inferential statistics refer to the output of repeated-measures *t*-tests. For each test, the degrees of freedom were 1001.

**Table S3**

*Summary of Mean Reliability Ratings in our Main Study*

| Headline Type | Pre-test | | Post-test | | *t* | *p* | *d*_z_ | *BF_10_* |
| --- | --- | --- | --- | --- | --- | --- | --- | --- |
|  | *M* | *SD* | *M* | *SD* |  |  |  |  |
| Conspiracy | 2.96 | 1.30 | 2.82 | 1.57 | 0.99 | .32 | 0.08 | 0.15 |
| Control | 4.67 | 1.26 | 4.61 | 1.27 | 0.46 | .64 | 0.04 | 0.10 |
| Discrediting | 3.35 | 1.31 | 3.25 | 1.50 | 0.72 | .47 | 0.06 | 0.12 |
| Impersonation | 3.21 | 1.30 | 3.25 | 1.43 | 0.34 | .74 | 0.03 | 0.10 |

*Note.* *M* and *SD* represent mean and standard deviation, respectively. Inferential statistics refer to the output of repeated-measures *t*-tests. For each test, the degrees of freedom were 149.

# Appendix D: Subset Analysis

**Table S4**

*Means and Standard Deviations of AUC and* B"*_D_* *Values for Participants Who Answered the “Trolling” Question Correctly*

| Test | AUC | | *B"*_D_ | |
| --- | --- | --- | --- | --- |
|  | *M* | *SD* | *M* | *SD* |
| Pre-test | .74 | .21 | .06 | .30 |
| Post-test | .73 | .23 | .09 | .35 |

# *Note*. *N* = 112.

# Appendix E: Supplemental Analyses of Stimulus Set and Training Effects

As reported in the main manuscript, we examined participants’ pre-test and post-test responses with the same set of stimuli. We achieved this by comparing Set A participants’ pre-test responses to Set B participants’ post-test responses, and vice versa. See Figures 1B and 1C of the main manuscript for the means and standard deviations for discrimination (AUC) and response bias (*B"*_D_) for each counterbalancing condition.

With respect to discrimination, a Welch two-sample *t*-test revealed that Set A participants’ pre-test AUC scores did not significantly differ to Set B participants’ post-test AUC scores, *t*(145.37) = 0.60, *p* = .55, *d* = 0.10, *BF*_10_ = 0.21. Likewise, a comparable analysis confirmed that Set A participants’ post-test AUC scores did not significantly differ to Set B participants’ pre-test AUC scores, *t*(147.62) = 0.06, *p* = .95, *d* = 0.01, *BF*_10_ = 0.18.

With respect to response bias, a Welch two-sample *t*-test revealed that Set A participants’ pre-test *B"*_D_ scores did not significantly differ to Set B participants’ post-test *B"*_D_ scores, *t*(142.51) = 0.61, *p* = .55, *d* = 0.10, *BF*_10_ = 0.21. A comparable analysis confirmed that Set A participants’ post-test *B"*_D_ scores did not significantly differ to Set B participants’ pre-test *B"*_D_ scores, *t*(143.34) = 0.83, *p* = .41, *d* = 0.14, *BF*_10_ = 0.24.

# References

Faul, F., Erdfelder, E., Lang, A.-G., & Buchner, A. (2007). G*Power 3: A flexible statistical power analysis program for the social, behavioral, and biomedical sciences. *Behavior Research Methods*, *39*(2), 175–191. https://doi.org/10.3758/BF03193146

Iyengar, A., Gupta, P., & Priya, N. (2023). Inoculation against conspiracy theories: A consumer side approach to India’s fake news problem. *Applied Cognitive Psychology*, *37*(2), 290–303. https://doi.org/10.1002/acp.3995
